# Rapid Inversion: Running Animals and Robots Swing like a Pendulum under Ledges

**DOI:** 10.1371/journal.pone.0038003

**Published:** 2012-06-06

**Authors:** Jean-Michel Mongeau, Brian McRae, Ardian Jusufi, Paul Birkmeyer, Aaron M. Hoover, Ronald Fearing, Robert J. Full

**Affiliations:** 1 Biophysics Graduate Group, University of California, Berkeley, California, United States of America; 2 Department of Bioengineering, University of California, Berkeley, California, United States of America; 3 Department of Integrative Biology, University of California, Berkeley, California, United States of America; 4 Department of Electrical Engineering and Computer Science, University of California, Berkeley, California, United States of America; 5 Department of Mechanical Engineering, University of California, Berkeley, California, United States of America; University of Vermont, United States of America

## Abstract

Escaping from predators often demands that animals rapidly negotiate complex environments. The smallest animals attain relatively fast speeds with high frequency leg cycling, wing flapping or body undulations, but absolute speeds are slow compared to larger animals. Instead, small animals benefit from the advantages of enhanced maneuverability in part due to scaling. Here, we report a novel behavior in small, legged runners that may facilitate their escape by disappearance from predators. We video recorded cockroaches and geckos rapidly running up an incline toward a ledge, digitized their motion and created a simple model to generalize the behavior. Both species ran rapidly at 12–15 body lengths-per-second toward the ledge without braking, dove off the ledge, attached their feet by claws like a grappling hook, and used a pendulum-like motion that can exceed one meter-per-second to swing around to an inverted position under the ledge, out of sight. We discovered geckos in Southeast Asia can execute this escape behavior in the field. Quantification of these acrobatic behaviors provides biological inspiration toward the design of small, highly mobile search-and-rescue robots that can assist us during natural and human-made disasters. We report the first steps toward this new capability in a small, hexapedal robot.

## Introduction

Fundamental laws of scaling in biology reveal why small animals have the opportunity to employ maneuvers that simply are unavailable to large animals. Turning ability and angular acceleration are largely dependent on rotational inertia [1], [2]. If we assume that animals are geometrically similar, then rotational inertia scales with body mass**^5/3^**. The rotational inertia of a 70 kg human is more than eight orders of magnitude greater than that of a 1 g insect, whereas their masses differ by only five orders of magnitude. In part, this explains why fruit flies during rapid saccades can execute a 90° turn in only 50 ms with 10 wingbeats [3]. Two centimetre long mother-of-peril caterpillars can recoil-and-roll backwards in 300 ms at 11 revolutions per seconds [4], [5]. Small lizards (3–6 g) running at high speed on artificial branches perform 90° turns with rotational velocities exceeding 600° per second [6]. Small geckos (3 g) can right themselves in mid-air in only 100 ms by a swing of their tail after falling upside down from the underside of a leaf [7]. Small geckos [8] and insects [9] can race up vertical surfaces at speeds near one meter per second (25 body lengths s^-1^) due to their advantageous strength to weight ratio [10], [11] and the ability of their feet to engage the surface. The number of asperities available for claw-based climbing scales inversely with the radius of a claw’s tip [12] allowing these animals to even run upside down [13]. These abilities conferred by physical scaling laws can provide key advantages to small animals, especially during escape responses from predators that demand rapid negotiation of complex environments challenging the fastest neural reflexes [14], [15].

Fortunately, these behaviors we admire in animals can now provide biological inspiration for small, mobile robots due to advancements in fabrication and rapid prototyping. Previously, microrobots with dimensions at the centimeter scale have been difficult to design using precision machining or microelectromechanical systems (MEMS) technology because of the difficulty in constructing high-strength segments with low-loss joints on the millimeter and micron scale. Now, new fabrication processes such as smart composite microstructures (SCM) [16] can integrate rigid links and large angle flexure joints through laser micromachining and lamination. Using these new rapid prototyping approaches, multiple design hypotheses of microrobots [17]–[19] and their parts can be tested and the unparalleled maneuverability we see in nature’s smallest animals can begin to be realized.

To understand the opportunities provided by scaling on the strategies used to maneuver in complex natural and human-made environments, we began by studying high-speed running in animals negotiating transitions in terrains. First, we ran the American cockroach *Periplaneta americana* at high speeds along an inclined track with a gap. To our surprise, the animal did not clear the gap, but exhibited a new behavior, a rapid inversion using its hind legs to swing underneath the ledge (Video S1). To explore the generality and biological relevance of high-speed transition strategies, we also studied geckos in the laboratory and in the field in the forest reserves of Singapore. We hypothesized that small animals running at high speed can attain an advantage in maneuverability by managing the transfer and redirection of kinetic and potential energy, so we compared the rapid inversion behavior to a passive pendulum model as a null hypothesis and then to a pendulum model with an initial velocity comparable to the animals. Our findings inspired the beginnings of a similar behavior in a physical model, a small hexapedal, prototype robot.

## Results

### Cockroaches

**Figure 1:**
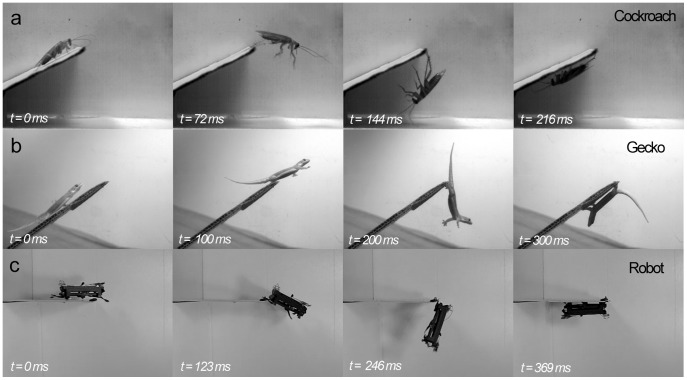
Sequence of rapid inversion behavior in a cockroach, gecko, and a robot prototype. Panels (*a*) and (*b*) show a high-speed 180-degree inversion behavior on an incline for cockroaches, *P. americana* and house geckos, *H. platyurus*, respectively. Panel (*c*) shows a cockroach-inspired hexapedal robot, DASH, successfully performing a similar maneuver from a horizontal platform with small Velcro hooks attached at the end of the hind legs.

**Figure 2:**
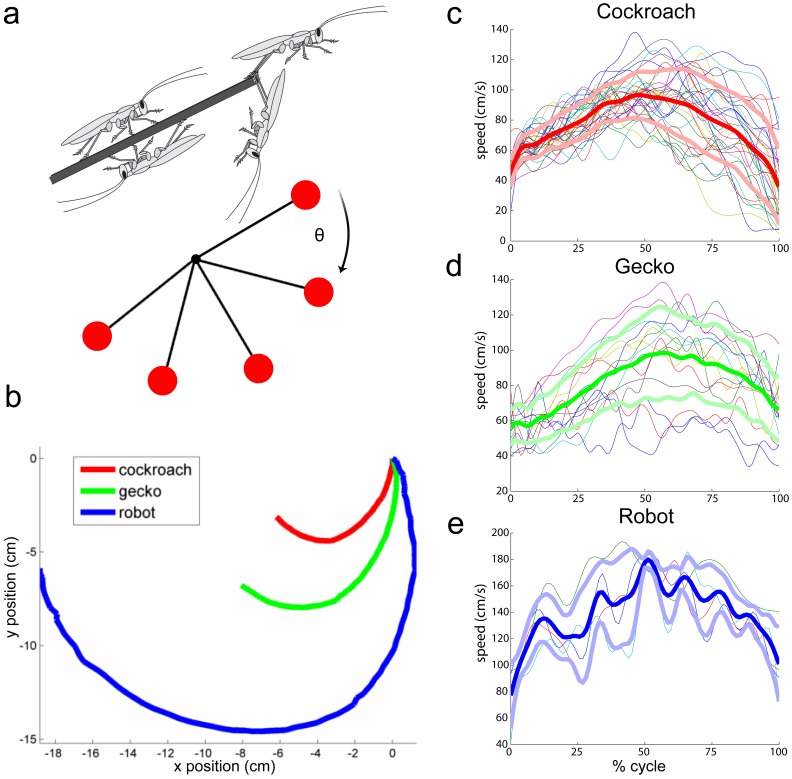
Kinematics of rapid inversion for animals and robot. In the top left panel (*a*) a representative pendulum-like model of the inversion behavior is shown swinging from rest until it contacts the underside of the ledge. The red circle represents the rostral or head position. In the bottom left panel (*b*), we plotted position data of the rostral region of the animals and robot with the initial position centered at the origin. In the three panels at the right, head speed data for the cockroach (*c*), gecko (*d*) and robot (*e*) are plotted as a function of scaled time (% cycle) to compare the speed profile across trials and animals. Bold thick lines show the average speed, whereas light thick ones show ±1 standard deviation. Thin lines in the background show the individual trial data for the animals and robot.

We discovered that American cockroaches (mass = 0.71±0.07 g, n = 6 animals, 32 trials) escaped at high speed (62.1±9.1 cm s^–1^) up an inclined track, dove forward and caught their tarsal claws, usually of both hind legs, on the substrate (Fig. 1a; Video S2). Data are reported as means ± s.d. unless otherwise noted. The claws briefly disengaged from the substrate as the tibia contacted the tip of the ledge, but then quickly reengaged at the tip [20]. With the claws attached, cockroaches executed a pendulum-like swing with peak head velocities of 109±12 cm s^–1^ (head angular velocities >1,200 deg s^–1^) around towards the underside of the substrate in only 127±22 ms (Fig. 2a-c). Cockroaches experienced mean peak accelerations of 3.8±0.9 Gs during the swing. After the swing, they secured their position and took several steps forward in an inverted posture. In approximately one quarter of the trials, cockroaches used a single leg to perform the maneuver without detectable changes in performance. Animals ran at full speed off the incline without requiring substantial braking. In only a few trials the animals continued to run off the ramp. Either slippage of the hind legs, loss of ground contact on the front and middle legs, or rapid change in pitch appeared to initiate the claw engagement reflex [20], suggesting active control. In all trials, the front and middle legs continued to cycle during the swinging maneuver. We attempted a balanced experimental design where we collected between 5 to 7 trials per individual.

To test whether claws are necessary for executing this behavior, we ablated the claws on the tarsi of both hind legs while leaving the sticky pads (arolium) intact. Using a paired comparison, we found that animals (n = 6 animals, 34 trials total with 5 to 9 trials per individual) with ablated claws failed 94% (32/34 trials) of the time at performing the maneuver while running at similar speeds (63.0±9.4 cm s^-1^) compared to the same animals with intact claws (*t*-test p = 0.76)(Video S3). In two trials, animals succeeded at performing the maneuver. For animals with ablated claws, we observed no change in strategy as the animals attempted to perform the same maneuver but failed.

### Geckos

The rapid inversion behavior was not unique to cockroaches. House geckos, *Hemidactylus platyurus,* (mass = 5.26±0.67 g, n = 5 animals, 17 trials) also running at high speeds (67.3±17.1 cm s^–1^) used a similar strategy as they rapidly approached the ledge (Fig. 1b; Video S4). During their dive, geckos engaged claws and sticky hairs (setae) near the tip of the ledge allowing them to swing around at high speeds (peak head velocity of 108±19 cm s^–1^, head angular velocities >900 deg s^–1^) towards the underside of the substrate in only 156±38 ms (Fig. 2d). Geckos experienced peak accelerations of 3.0±0.8 Gs during the swing. We attempted a balanced experimental design where we collected between 2 to 4 trials per individual animal.

**Figure 3:**
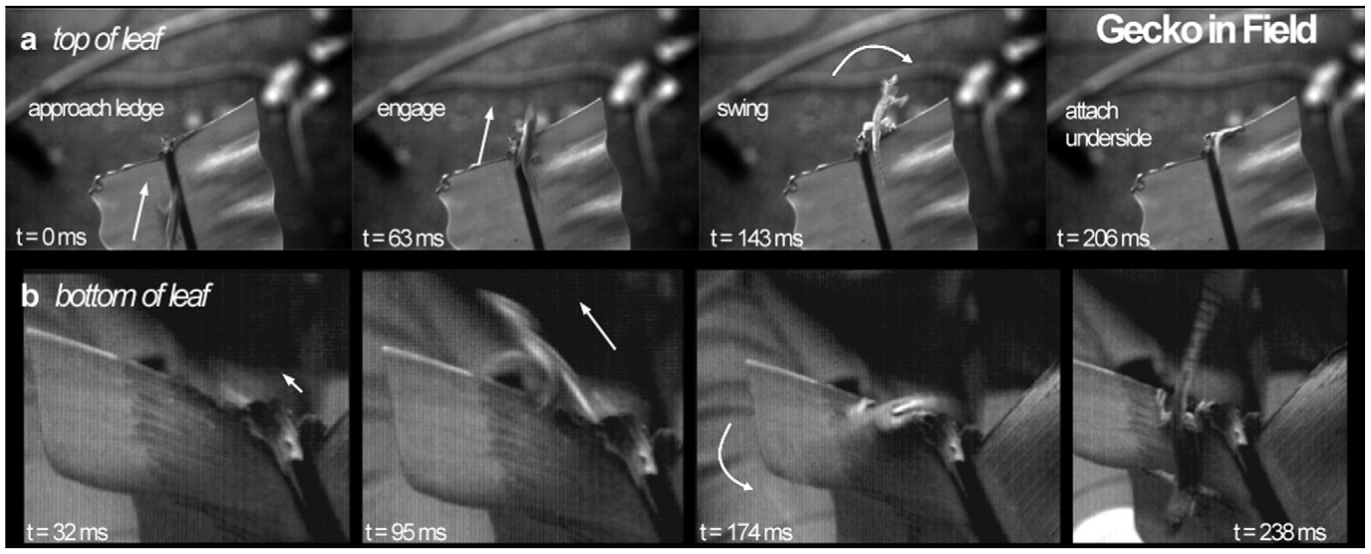
Flat-tailed house gecko, *H. platyurus* in its native environment in the rainforests of Singapore. The two panels show a sequence of the inversion behavior from the top (*a*) and bottom (*b*) of a fern leaf recorded in the field with high-speed videography. After moving over the robust parts of the fern leaf with a rigid midrib beneath that supported their body weight, the gecko engaged its claws near the tip of the leaf and performed a pendulum-like swing towards the underside.

We observed the rapid inversion behavior in the gecko’s natural environment, the forests of Singapore (Fig. 3). Geckos (mass = 3.75±0.4 g) ran over the lamina of ferns and stretched their forelimbs outwards as their torso went over the leaf’s edge (Video S5). We studied eleven animals and we observed the behavior in three animals. They anchored their rear legs within the blade of the fern, thus causing the body to swing around towards the underside of the leaf as a result of their inertia. The behavior was analogous to the discovery made in the laboratory and demonstrates its potential effectiveness in traversing the animal’s native habitat.

### Robot Prototype

The novel rapid inversion behavior can provide initial inspiration for the development of new capabilities in running robots (Fig. 1c). Using the cockroach-inspired hexapedal robot DASH (Dynamic Autonomous Sprawled Hexapod) [19], we simulated claw action by attaching a pad of Velcro hooks on the hind legs. We glued the “loop” side of the Velcro on the substrate near and underneath the ledge. DASH (mass = 16.0 g, n = 4 trials) ran at high speed along a horizontal track (88.0±3.5 cm s^–1^) because at this early stage in design inclines resulted in slipping. DASH successfully swung around toward the underside of the track in only 221±43 ms (“head” angular velocity >600 deg s^–1^) and then stuck to the ledge and underneath the track (Fig. 2e; Video S6). Robots were exposed to peak accelerations of 5.2±0.12 Gs during the swing.

### Pendulum Model

**Figure 4:**
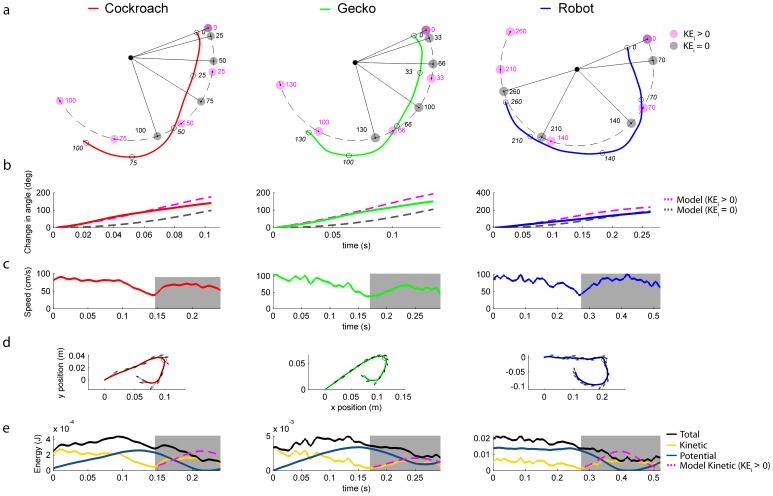
Comparisons of animal and robot kinematics to a pendulum model. Panel (*a*) compares a pendulum model without transfer of kinetic energy (KE = 0; grey bob) and with complete transfer of kinetic energy (KE>0; magenta bob) to the animal and robot trajectories as a function of time (ms) from representative position data from the COM of the cockroach (red), gecko (green), and robot (blue). The pendulum base joint represents the average position of the feet during the maneuver. The cockroach and gecko started swinging at an angle of approximately 30 degrees from the body long axis relative to the horizontal, whereas the robot initiated swinging near the horizontal relative to the body long axis (0 degree). Panel (*b*) shows the change in angle relative to the initial angle at the start of the swing for animals and robot compared to our two models. Panel (*c*) shows the speed of the animals and robot. The grey area represents the period of swinging defined as the point of slowest speed following foot engagement until all legs contacted the underside of the ledge. Panel (*d*) shows the position of the COM of the animals and robot during the complete rapid inversion maneuver for a representative trial. Arrows indicate the resultant velocity vectors (m s^–1^) at intervals of 20 ms. The black open circle indicates the region where the speed is slowest. Panel (*e*) shows the corresponding energy profiles. The grey area represents the same period as defined in (*c*) above. The dashed curve in magenta shows the total kinetic energy for the pendulum model if transfer were complete.

We tested the hypothesis that animals swung around to the underside of the ledge like a pendulum by first comparing the swing kinematics to a physical pendulum model with zero transfer of kinetic energy as a null hypothesis. We determined the parameters of the pendulum model using estimates of morphology and matching the initial conditions to the animal or robot positions (see Methods). If we assume that the body or center of mass of the animal or robot represents the bob of a pendulum subject to only gravitational force and starting from rest with no added kinetic energy, then we can trace the trajectory from the time the animal engages its claw or the robot sticks until the swing underneath the ledge is complete (Fig. 4a; grey circles).

For both the cockroach and gecko, we found significant differences in body angle when comparing the animals and model at different time intervals in the full cycle (Fig. 4a,b). Here we define a full swing cycle from when the animal reaches a minimum velocity in transition from running to swinging (see Fig. 4c–d) until all legs come in contact with the underside of the ledge. For example, the cockroach is approaching a near vertical orientation with respect to the body long axis in the swing at half cycle (50 ms; Fig. 4a red line) due to its more rapid initial velocity (Fig. 2c), while the passive pendulum model is just moving down from a horizontal position after the same time period. Therefore, we reject our null hypothesis that a passive pendulum with no transfer in kinetic energy explains the swing kinematics of the animals.

The rapid inversion behavior better approximates a pendulum model with initial kinetic energy (Fig. 4a). In fact, we found no evidence that the animals reached a velocity of zero in transitioning from inclined running to swinging (see Fig. 4c–d). To test whether animals swing like a pendulum with an initial velocity, we set dθ/dt equal to the angular velocity at the transition between running and swinging where speed was lowest using estimates from experimental data (Fig. 4b). We found that in the first half of the swing cycle, the pendulum model with an initial velocity accurately predicts the trajectories of both the cockroach and gecko suggesting that the animals may conserve energy in the transition from running to swinging. To quantify the possible conservation, we estimated the total energy transfer in the transition from running to initial swinging (see Methods). Total energy transfer for cockroaches (n = 5 trials) and geckos (n = 3 trials) was 78.1±10.0% and 74.4±5.6%, respectively. The DASH robot prototype (n = 3 trials) transferred 72.5±13.5% of its energy.

## Discussion

As first articulated by Galileo in *Two New Sciences*, scaling in biology can impose important constraints on performance as well as permit new opportunities. Non-linear physical forces governing movement can shape an animal’s behavioral repertoire. The novel behavior we have discovered in small animals demonstrates an important effect of scale, specifically the scaling of rotational inertia and claw-substrate interactions that allow these animals to outmaneuver the best human acrobats. Using a pendulum-like swing with their hind legs as grappling hooks, these small, legged runners represent another example of using the natural dynamics of the body and appendages to effectively complete a maneuver (Fig. 1) [6], [7], [21]. The rapid inversion behavior may provide important advantages for predatory avoidance by rapid disappearance in natural settings, as demonstrated by our study of wild geckos in South East Asia (Fig. 3). Moreover, the new capability may provide insights into how animals can negotiate complex environments requiring transitions that demand rapid transfer and redirection of energy [22]. The study of high-speed transitions including, but not limited to inclines, gaps, and landing maneuvers represents a frontier in biomechanics research that will enable the unravelling of new principles behind rapid dynamic reconfiguration of bodies and appendages.

### Comparison to Simple Pendulum Model and Brachiation

To test the hypothesis that cockroaches and geckos configure their legs and bodies during rapid inversion to swing like a pendulum, we applied a template based on pendulum dynamics that has been used to understand cyclic animal movements such as walking, running and brachiating. Inverted pendulum models for walking capture the exchange between kinetic and potential energy, as well as the collisions and energy redirection [23], [24]. This is true not only for their applications to bipeds, but models for walking crabs [25] and lizards [26] show patterns consistent with pendular energy exchange reaching 51–55%. In insects, pendular exchange has been proposed for the suspensory or bridging locomotion of spiders that walk upsidedown [27]. When landing, house flies and honeybees rely on energy redirection to swing their bodies to initiate touchdown [28], [29]. Similarly the blue-winged grasshopper uses its legs to redirect its body 180 degrees to perform a “hook” landing [30]. Given the novelty of the rapid inversion behavior, we only can compare it to a well-studied swinging behavior, brachiation. Gibbons display two types of brachiation: continuous contact, similar to walking, and ricochetal, analogous to running that has a flight phase [31]. Even using the simplest possible model for a single swing, a point mass with a massless support arm, both brachiation gaits display substantial pendular exchange between kinetic and potential energy [32]. Applying a physical pendulum model with zero transfer of kinetic energy to rapid inversion revealed substantial deviations from the actual trajectories in both the cockroaches and geckos, especially at the onset of the swing (Fig. 4a). The same pendulum with nonzero initial kinetic energy better represented the beginning portion of the swing cycle of both animal trajectories suggesting that energy is effectively transferred from running to swinging, but not without losses (Fig. 4a: see time 75 ms and 100 ms for the cockroach and gecko, respectively). Our results suggest that approximately 20% of the total energy is lost in the initial transition based on calculations of total energy transfer at the COM (Fig. 4e).

A next step in modeling rapid inversion can be guided by the efforts to study brachiation. A fundamental feature of brachiation gaits is the minimization of collisional energy loss due to discontinuities in the center of mass trajectory. In the case of rapid inversion, it is also likely that energy losses occur due to a discontinuity in trajectory, particularly during the transition from running to swinging which requires a redirection of the available kinetic energy. The trajectories of animals studied here, although more rapid than a passive pendulum, lag behind a pendulum model near the end of the cycle even when given an initial velocity (Fig. 4a). Losses likely occur due to damping in the legs by muscles and joint membranes during the swing. At the same time, we might consider the possibility that the legs store and return energy like springs as observed during running gaits modeled by a spring-loaded inverted pendulum [24]. More representative brachiation models include multiple links that approximate the gibbon’s head, torso and legs [33]. These models account for rotational kinetic energy and its effects. Our kinematic data show the possibility that the kinetic energy of running can be transferred to rotation of body segments (Video S2) and appendages such as a tail (Video S3). Much of brachiation modeling has focused on energy [32], [33] and reasonable passive models show the possibility of very low energetic cost. By contrast, more costly active brachiation using muscle power appears to have the advantage of recovering from perturbations in the natural environment using neural feedback [31]. Given that rapid inversion is not a repeated, sustained activity like brachiation, we hypothesize that energy saving is less important, whereas effective transfer of energy to complete the behavior as quickly as possible with a sufficient level of stability is paramount.

### Neuromechanical Control

Future investigation of the stability and control of rapid inversion and similar acrobatic behaviors will uncover the role of active neural sensory adjustments versus the feedforward, passive dynamics we model here. During the transition from running to swinging, we observed that the middle and front legs of cockroaches continue to cycle in free air, while the hind legs remain attached to the ledge via claw engagement. Leg cycling was not observed in geckos, thus suggesting alternate control responses in a vertebrate of similar size. In the cockroach, we noted that front and middle legs continued to cycle out of phase in free air much like in an alternating tripod gait used for high-speed running. This suggests that cockroaches could complement the task-level feedback required for claw engagement with a feedforward mode during this high-speed behavior. Pattern generators providing signals to the muscle controlling limbs have a flexible control architecture capable of decoupling the action of individual or pair of legs consistent with studies in other insects moving more slowly [34].

### Bio-inspiration

We used the rapid inversion behavior to inspire the initial design of a legged robot named DASH that begins to demonstrate this new level of maneuverability (Fig. 1c, 2e, 4 Robot). The preliminary design may not be as effective as the animals in redirecting energy into the swing as evidenced by the losses later in the cycle (Fig. 4b), but given the flexible manufacturing approach available [16], [19], future adjustments are possible. We already are developing several active and passive, bio-inspired claw designs to replace the prototype Velcro hooks. The new behavior emphasizes a major difference that remains between animals and our best robots. We have designed robots that can run or climb, but few can do both and effectively transition from one surface to another. We anticipate that the quantification of acrobatic behaviors in small animals will continue to provide biological inspiration resulting in small, more highly mobile sentinel and search-and-rescue robots that assist us during natural and human-made disasters.

## Materials and Methods

### Laboratory Animal Husbandry and Ethics Statement

Adult male American cockroaches, *Periplaneta americana*, were acquired from a commercial vendor (Carolina Biological Supply Company, Burlington, NC, USA) and housed in plastic cages maintained at a temperature of 27°C. Cockroaches were exposed to a L:D cycle of 12 h:12 h and given fruits, dog chow and water *ad libitum*. Flat-tailed house geckos, *Hemidactylus platyurus*, used in the laboratory were purchased from commercial vendors (The Reptile Company, Endicott, NY; Glades Herp, FL; California Zoological Supply, Los Angeles, CA). Note that this is the species used in climbing experiments, but now resides in a different taxa [35]. Geckos were housed in common cages in an animal care facility at UC Berkeley. They were fed with a diet of crickets with vitamin and mineral supplements as deemed suitable by veterinarians. Water was provided *ad libitum*. Geckos were kept in an environmental control room with 12 h of light per day and at a temperature of 25±2°C. Trials were conducted at an average temperature of 30°C. The Animal Care and Use Committee at the University of California, Berkeley, whose activities are mandated by the U.S. Animal Welfare Act and Public Health Service Policy, approved all experimental procedures described for these research projects.

### Track

We ran cockroaches and geckos at high speeds along an inclined track in an arena enclosed with Acrylic walls. For both species, the track abruptly ended. The track was inclined at approximately 30^o^ and ended with a ledge whose undersurface matched the track substrate (cardboard for cockroach and cardboard with a thin layer of 40-grit sandpaper for geckos). Animals started from the bottom of the track and upon eliciting an escape response, they ran up the inclined track.

### Animal and Robot Kinematics in the Laboratory

We captured high-speed videos of the animals using two cameras (X-PRI, AOS Technologies) positioned at the top and the side of the arena. The cameras were synchronized and recorded at 500 frames per second. For the cockroaches and geckos, we tracked a 2-dimensional projection of the head and feet attachment positions to obtain kinematic measurements of the maneuver (ProAnalyst, Xcitex, Inc.). All digitized data were filtered and analyzed with custom scripts (Matlab, MathWorks). All animal kinematic data used to calculate velocities and accelerations were low-pass filtered using a 2^nd^ order Butterworth filter with a cut-off frequency of 75 Hz, which was approximately three times the stride frequency of *P. americana* for the recorded running speeds. For the robot, we used a low-pass filter at 50 Hz which was also about three times stride frequency. To estimate the center of mass (COM) of *P. americana*, we manually tracked the head and caudal region of the abdomen to define the body length and determined the COM to be 50% of body length based on previous measurements [36]. For the gecko, we manually tracked the rostral and caudal region of the torso and took the COM to be between these points. We video recorded the robot, DASH, performing the maneuver using high-speed videography at 300 frames per second (Casio Exilim EX-F1) and tracked its motion for kinematic analysis (ProAnalyst, Xcitex, Inc.). For the robot, we manually tracked the front and back region to define the body long axis and determined the COM to be at 50% of body length. Speeds shown in Figure 2 were calculated using the rostral portion of the body for animal and robots, whereas velocities shown in Figure 4 are for the COM.

### Claw Ablation Experiment

To study the role of claws, we performed claw-ablation surgeries on cold-anesthetized cockroaches. The pair of claws on each metathoracic leg was ablated under a microscope using fine dissection scissors (Fine Science Tools). Care was taken to leave the membranous base, the arolium, intact. Animals were allowed at least 24 h to recover after the surgery. We then ran the animals with the same procedure as controls.

### Field Behavior

To establish the generality of our results on the incline running and ledge climbing performance of *H. platyurus* from the laboratory, we sought to relate it to the animal’s ecology and natural history by conducting field research in the context of a South-East Asian lowland tropical rainforest habitat. For the field experiments, we used eleven wild-caught geckos (mass = 3.75±0.4 g).

The National Parks Board of The Republic of Singapore approved a request to study the locomotion of geckos with a field research permit and the Wildlife Reserves Singapore allowed us to capture house geckos *H. platyurus* (Specimen Collection Permit # NP/RP955B). We accommodated lizards in portable terraria with ambient humidity (ca. 85%) and temperature (ca. 38°C) for the shortest duration possible at our field site in the Wildlife Reserves. As a medium for our experiment, we selected the “Bird’s Nest Fern” *Asplenium nidus* which is native to the lowland tropical rainforest. It can be observed at great heights above ground and therefore lives in the same canopy habitat as *H. platyurus*. The mid-rib of this fern renders the overall leaf very rigid and capable of supporting the weight of *H. platyurus*.

We used three digital video cameras (X-PRI, AOS Technologies) to capture simultaneously dorsal, sagittal and cranial views of the lizards running on the Bird’s Nest Fern. Video frames from all three camera views were synchronized. The data were stored on a portable computer. The lighting conditions in the field can change rapidly due to patchy clouds temporarily obscuring the path of the sunlight. We responded to this situation by working with the lighting available and recorded at different frame rates ranging from 32–500 frames per second. We frequently adjusted the aperture on one 50 mm and two 25 mm lenses to ensure an adequate amount of light be made available to the high-speed video cameras in face of the dynamically changing lighting conditions.

To acquire individual morphometrics, the house gecko’s length was measured while holding the animal in hand, whereas the mass was determined by placing the animal on a small scale. All animals were released at the region of capture after video recording. No harm was done to the animals or surrounding landscape during this process. All animals were released shortly after capture if they were deemed not suitable for the study. No detrimental effects resulted from the short confinement. All methods of capture and handling are well-established standard techniques used by herpetologists. Geckos were not recaptured.

### Pendulum Model

We determined the pendulum model trajectory by solving a nonlinear physical pendulum model of the form
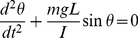
(1)using the *ode45* solver in Matlab (Mathworks) where *θ* is the angle as defined in Figure 2a, *m* is the mass of the animal or robot, *g* is the acceleration due to gravity, *L* is the pendulum length and *I* is the moment of inertia. We determined the pendulum length *L* by computing the average length from feet to center of mass during the swing of the animals and robot. By the parallel axis theorem, the moment of inertia *I* of the animals and robot was approximated as
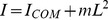
(2)where *Icom* is the moment of inertia at the COM and *mL^2^* is the parallel axis term. The moment of inertia of the animals and the robot was approximated as an ellipsoid and flat rectangular plate, respectively. Since the moment of inertia of the legs in the animals and robot were less than 5% of the parallel axis term, they were not included in the final calculations.

This model has two initiation conditions: initial angle 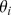 and initial angular velocity 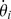. We set the initial conditions such that the angle of the pendulum 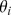 matched the initial angle of the animals and robot. We calculated 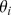 by taking the angle between a vector defined by the point at the end of the ledge where the feet attached to the COM and a vector defined by the horizontal axis parallel to the ground (Fig. 2a). We defined the initiation of the swing maneuver as the time when the COM attained minimum speed (Fig. 4). For the model with no initial kinetic energy, we set 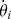 equal to zero. For the pendulum model with initial kinetic energy, we set 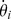 equal to
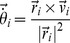
(3)where 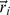 is the vector from the ledge to the COM and 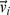 is the velocity at the transition from running to swinging. We estimated 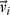 using experimental data (see Fig. 4c). To compare the progression of the animals and robot in Figure 4, we linearly interpolated the animal and robot position data to attain a temporal resolution of 0.5 ms. We assumed aerodynamic drag forces to be negligible as calculations using a flat plate model perpendicular to flow yielded drag forces less than 10% for cockroaches and geckos (Appendix S1).

To measure the total energy transfer in the transition from running to swinging, we selected trials in which cockroaches used both feet to engage at the ledge and continued engaging both feet during inversion. These trials had very little out-of-plane motion, thus improving our estimate of the 2D-projected COM position. For geckos, we also selected trials in which the animal ran off the ledge with both feet engaged. In addition, we rejected trials in which the ventral region of the abdomen contacted the ledge which could contribute to energy losses due to friction. For the robot, we selected three trials out of four in which both feet remained attached to the Velcro pad for the longest period during inversion to minimize out-of-plane motion.

### Robot Assembly

The cockroach-inspired DASH robot was used as the platform to recreate the claw action observed in the legged animals. The hexapedal robot is 10 cm long and has a mass of 16 g with onboard electronics, actuation, and battery. It was constructed using the cardboard Smart Composite Microstructures manufacturing process which produces folded structures from rigid cardboard beams and polymer flexure joints. Velcro was bonded to compliant pads that were attached to the hind feet of the robot cantilevered in the aft direction. The Velcro enabled the robot to gain purchase at the edge of the substrate while the compliant pads provided the necessary degree of freedom to allow the robot to rotate to the inverted position. Velcro on the underside of the substrate, paired with matching Velcro bonded directly to the front feet of DASH, allowed the robot to perch inverted beneath the substrate after the inversion maneuver.

## Supporting Information

Video S1 **A top view of a cockroach,** ***P. americana,*** **performing a high-speed inversion while running up a ramp.** The first sequences are real time. The second sequences are slowed 10X.
(MOV)

Video S2 **Side view of a cockroach,** ***P. americana,*** **performing a high-speed inversion while running up a ramp.** The first sequences are real time. The second sequences are slowed 10X. The last sequence is slowed 50X.
(MOV)

Video S3 **Side view of a cockroach** ***P. americana*** **attempting to perform an inversion after claw ablation, but failing.** The first sequences are real time. The second sequences are slowed 10X. The last sequence is slowed 50X.
(MOV)

Video S4 **Side view of a house gecko,** ***H. platyurus,*** **performing a high-speed inversion while running up a ramp.** The first sequences are real time. The second sequences are slowed 10X.
(MOV)

Video S5 **A wild-caught gecko,** ***H. platyurus,*** **performs a high-speed inversion while running on a leaf in the rainforest of Singapore.** Left panel shows bottom view and right panel show top view. The first sequences are real time. The second sequences are slowed 10X.
(MOV)

Video S6 **Robot (DASH; Dynamic Autonomous Sprawled Hexapod Robot) running at high-speed performing rapid inversion while running near a ledge.** The first sequences are real time. The second sequence is slowed 12X.
(MOV)

Appendix S1 (DOC)
