# Supplementary material for: Rapid Inversion: Running Animals and Robots Swing like a Pendulum under Ledges
Source: PLoS One. 2012 Jun 6;7(6):e38003. doi: 10.1371/journal.pone.0038003 (PMC3368944; doi:10.1371/journal.pone.0038003)
Supplement: Appendix S1 — (DOC) [file pone.0038003.s007.doc]

**Appendix S1**

If we model a cockroach as a rectangular flat plate perpendicular to an air-flow, the drag force is

$$F_{d}=\frac{1}{2}\rho v^{2}C_{d}A$$

where *F_d_* is the drag force, *ρ* is the density of the medium, *v* is the velocity of the object relative to the air flow, *A* is the cross-sectional area and *C_d_* is the drag coefficient. A cockroach during inversion will experience peak Re numbers of

$$\mathrm{Re}=\frac{Ul}{\upsilon}=\frac{\left(1.10ms^{-1}\right)\left(0.035m\right)}{0.000015m^{2}s^{-1}}\approx3.0\times10^{3}$$

where *l* is the body length (estimated for *P*. *americana*), *U* is the velocity of the air relative to the animal (average peak velocity of 1.10m s^-1^, see Results) and υ is the kinematic viscosity of the fluid (15 x 10^-6^ for air at 20^o^C). Since Re < 10^5^, we assume that the flow is laminar.

For a flat plate perpendicular to flow in the laminar regime with aspect ratio < 0.1, the drag coefficient is ~ 2.0 [1]. Assuming air density at 20^o^C, we can calculate the peak drag force using estimates of cockroach morphologies as follows:

$$F_{d}=\frac{1}{2}\rho v^{2}C_{d}A=0.5\left(1.2kgm^{-3}\right)\left(1.10ms^{-1}\right)^{2}\left(2.0\right)\left(0.035m\times.01m\right)\approx.050mN$$

which is approximately 7.0% of body weight for a cockroach of .71g (average mass, see Results).

For geckos, we can apply the same calculation. We find that geckos experience peak Re numbers of

$$\mathrm{Re}=\frac{Ul}{\upsilon}=\frac{\left(1.10ms^{-1}\right)\left(0.054m\right)}{0.000015m^{2}s^{-1}}\approx4.0\times10^{3}$$

using morphological data from Jusufi et al.

Thus, we find that the flow regime is laminar. Again, using the morphologies from Jusufi et al. and assuming that the tail has negligible drag compared to body drag, we can calculate the drag forces using the same the same flat plate model as the cockroach,

$$F_{d}=\frac{1}{2}\rho v^{2}C_{d}A=0.5\left(1.2kgm^{-3}\right)\left(1.10ms^{-1}\right)^{2}\left(2.0\right)\left(0.054m\times0.037m\right)\approx2.9mN$$

which is approximately 6.0% of body weight for a gecko with a mass of 5.26g (average mass, see Results) similar to that estimated in Jusufi et al.

**References**

1. Munson, BR, Young, DF, Okiishi, TH (2006) Fundamentals of Fluid Mechanics (Fifth Edition). *John Wiley & Sons.*

2. Jusufi, A, Kawano, DT, Libby, T, Full, RJ (2010) Righting and turning in mid-air using appendage inertia: reptile tails, analytical models and bio-inspired robots. *Bioinspir Biomim* 5: 1-12.
